# BDH1 acetylation at K116 modulates milk fat production in dairy goats

**DOI:** 10.21203/rs.3.rs-7156146/v1

**Published:** 2025-07-29

**Authors:** Tiantian Xiong, Chong Chen, Xinglong Gong, Chengming Han, Min Tian, Jun Luo, Lu Deng, Juan J. Loor, Cong Li

**Affiliations:** Northwest Agriculture and Forestry University: Northwest A&F University; Northwest Agriculture and Forestry University: Northwest A&F University; Northwest Agriculture and Forestry University: Northwest A&F University; Northwest Agriculture and Forestry University: Northwest A&F University; Northwest Agriculture and Forestry University: Northwest A&F University; Northwest Agriculture and Forestry University: Northwest A&F University; Northwest Agriculture and Forestry University: Northwest A&F University; University of Illinois Urbana-Champaign; Northwest A&F University

**Keywords:** acetylome, modification sites, BDH1 protein, fat biosynthesis, dairy goat

## Abstract

**Background:**

Goat milk is increasing valued for its superior nutritional profile, digestibility, and unique compositional properties. Protein acetylation, a pivotal post-translational modification, plays a critical role in the regulation of biosynthesis and metabolic processes. This study aims to identify key acetylated proteins and their modification sites governing milk production and the synthesis of milk components in dairy goats. Our findings establish a mechanistic foundation for elucidating molecular regulation of lactation and enhancing milk quality through targeted breeding strategies.

**Results:**

The acetylome profile of mammary gland tissues in dairy goats was successfully established. A total of 862 significantly acetylated proteins were identified across two lactation phases, and a total of 2,028 acetylation modified sites were identified in mammary gland tissues in dairy goats. Differentially acetylated proteins were predominantly localized in the cytoplasm (39.98%). From these, 54 key acetylated proteins, including MTOR, BCAT2, QARS1, GOT1, GOT2, BDH1, ACSS1, STAT5B, FABP5, and GPAM were identified as candidates potentially involved in milk protein synthesis, milk lipid synthesis, lactose synthesis, and other lactation-related biological processes in dairy goats.

Among them, the acetylation modification of the β-hydroxybutyrate dehydrogenase 1 (BDH1) protein was characterized in dairy goats. The HDACs family was identified as primary regulators mediating the deacetylation of BDH1. Acetylation of BDH1 promoted the expression of *LXRα, ACSL1* and *SCD1*genes, while its deacetylation induced the expression of *SCD1, FASN* and *ACSL1* genes. BDH1 acetylation/deacetylation significantly reduced the expression of the *SREBP1* gene. Furthermore, BDH1 acetylation promoted the formation of lipid droplets and the synthesis of triglycerides in mammary epithelial cells of dairy goats (GMECs).

**Conclusions:**

This study established, for the first time, the comprehensive acetylome of mammary gland tissue in dairy goats, revealing a substantial number of differentially acetylated proteins and modification sites. We demonstrate that acetylation of BDH1—regulated by HDACs—promotes lipid droplet biogenesis and triglyceride synthesis in GMECs through transcriptional modulation of key lipogenic genes (*LXRα, ACSL1, SCD1, FASN*) and suppression of *SREBP1*. These findings provide novel mechanistic insights into the post-translational regulation of mammary lipid metabolism during lactation.

## Introduction

With the growing emphasis on healthy diets, global demand for goat milk and its products continues to rise, largely due to their favorable nutritional properties, including high digestibility, low allergenicity, and a rich content of short-chain fatty acids (SCFAs) [1–4]. To meet the quality standards required for premium dairy products such as infant formula [5], it is essential to enhance the protein and fat content of goat milk, particularly during the peak lactation period, in order to produce “high-yield, high-quality” milk. A comprehensive understanding of the molecular mechanisms regulating milk protein and fat synthesis is therefore critical to improving the overall quality of goat milk.

Post-translational modifications (PTMs) serve as evolutionarily conserved regulatory mechanisms that modulate protein activity, stability and localization, thereby ensuring proper protein function and enabling the precise regulation of metabolic pathways and biological processes in organisms [6, 7, 8]. Among these, lysine acetylation plays a core role and has been shown to directly regulate glycolysis, fatty acid metabolism, and other metabolic pathways involved in the synthesis of milk components [9, 10]. Preliminary studies in mammary tissues suggest that acetylation may be involved in the regulation of lactation. For instance, sodium butyrate has been shown to significantly increase histone H3 acetylation levels and upregulate β-casein gene expression in bovine mammary epithelial cells by inhibiting histone deacetylase (HDAC) activity [11]. Similarly, sodium propionate inhibits HDAC activity by enhancing histone acetylation, thereby affecting mammary cell metabolism [12]. However, most existing studies have mainly focused on the indirect effects of histone acetylation, while the regulatory roles of non-histone acetylation modifications, particularly in milk proteins and key enzymes involved in milk lipid synthesis, remain largely unknown. Furthermore, there is significant research gap regarding these mechanisms in dairy goats, an economically important livestock species.

In recent years, proteomics technologies based on affinity enrichment coupled with high performance liquid chromatography-tandem mass spectrometry (HPLC-MS/MS) have emerged as powerful tools for revealing key acetylation-modified proteins involved in specific physiological and pathological processes. For example, this approach has been successfully used to map differentially expressed acetylated proteomes in left ear tissues of patients with chronic atrial fibrillation compared to those in sinus rhythm [13, 14]. Similarly, Yao integrated transcriptomic, proteomic, and acetylomic analyses to elucidate the regulatory roles of acetylation modifications in theanine biosynthesis in tea plants [15]. Despite these advantages, the global profiling of lysine acetylation modifications in the lactating mammary gland of dairy goats and their regulatory functions in milk protein and milk lipid synthesis remains largely unexplored. Therefore, this study aims to perform comprehensive proteomic and acetylomic analyses of mammary gland tissues from dairy goats during the dry and lactation periods using HPLC-MS/MS technology. The objective is to identify key acetylated proteins and specific modification sites that affect the synthesis of milk components. Subsequently, selected candidate targets were functionally validated using a dairy goat mammary epithelial cell model to elucidate the molecular mechanisms by which lysine acetylation modifications regulate milk component biosynthesis.

## Materials and methods

### Sample collection

Based on the known negative correlation between milk yield and milk protein and fat content traits across the lactation period, six healthy Saanen dairy goats from the same litter with similar production levels were selected from the dairy goat breeding farm at Northwest A&F University. Three goats were in the peak lactation period (day 60 of lactation) and three were in the dry period. Mammary gland tissues were collected via surgical methods while the goats were alive. All experimental procedures were performed in accordance with the guidelines approved by the Institutional Animal Care and Use Committee (IACUC) of Northwest A&F University, China (Approval No. DK2021054). Mammary gland tissues were immediately frozen into liquid nitrogen and further stored at −80°C until further analysis.

### Total protein extraction and acetylation assay

Mammary gland tissue samples stored at −80°C were pulverized in liquid nitrogen-cooled mortars. Powdered tissues were lysed in buffer (8 M urea, Sigma-Aldrich, USA; 1% protease inhibitor, Merck Millipore, Burlington, MA, USA; 3 μM TSA, Trichostatin A, MedChemExpress, Shanghai, China; 50 mM NAM, Nicotinamide, Sigma-Aldrich, USA; 2 mM EDTA, Ethylenediaminetetraacetic acid, Sigma-Aldrich, USA), and the supernatant was collected by centrifugation for 10 min at 12,000xg at 4°C after sonication. Protein concentration was determined using a BCA kit (#23225; Thermo Fisher Scientific, MA, USA), and sample homogeneity and stability were verified by Caulphas Brilliant Blue staining (Sigma-Aldrich, USA). After 20 μg of protein was taken for 12% SDS-PAGE electrophoresis and transferred to a PVDF membrane (Merck Millipore, MA, USA), it was incubated with an anti-acetylated lysine antibody (1:1,000,CST, MA, USA) and an HRP-labeled secondary antibody (1:4,000, CWBIO, Beijing, China), and detected using the ECL Chemiluminescent Reagent (Thermo Fisher Scientific, USA) was developed on a QuickChemi 5200 Chemiluminescent Imaging System (Monad Biotech, Wuhan, China) to detect bands of different molecular weight acetylated proteins.

### HPLC-MS/MS analysis and database searches

Protein solutions were reduced by adding dithiothreitol (DL-Dithiothreitol, DTT) (Sigma-Aldrich, USA) to a final concentration of 5 mM for 30 min at 56°C. Subsequently, iodoacetamide (IAM) (Sigma-Aldrich, USA) was added to a final concentration of 11 mM and incubated for 15 min at room temperature and protected from light. The samples were incubated at room temperature away from light for 15 min. Finally, the urea concentration of the samples was diluted to less than 2 M. Trypsin (Promega, WI, USA) was added at a ratio of 1:50 for overnight digestion at 37°C. The samples were incubated for 30 min at room temperature away from light. Trypsin was added at a 1:100 mass ratio (trypsin: protein) for another 4 h. Peptides were desalted and lyophilized by Strata X C18 (Phenomenex, CA, USA), labeled with Tandem Mass Tag (TMT) reagent (Thermo Fisher Scientific, USA), and then treated with acetylated antibody resin (PTM Biolabs, IL, USA) enrichment, eluted with 0.1% TFA trifluoroacetic acid (Sigma-Aldrich, USA), and finally analyzed by LC-MS/MS (Q Exactive HF-X; Thermo Fisher Scientific, USA), data were analyzed by MaxQuant (v1.5.2.8) for retrieval.

### Proteomics and acetylomics analysis

The Eukaryotic Homologous Group (KOG) database was selected as the comparison database, and the identified acetylated proteins were analyzed by BlastX comparison, and then functionally classified according to the annotation terms of KOG (counting the number of acetylated proteins in each KOG functional class). GO annotation was performed using the UniProt-GOA database (http://www.ebi.ac.uk/GOA/). Acetylated protein ID numbers were entered into the database to be converted to UniProt ID numbers, which were next mapped to GO ID numbers to classify them according to three categories: biological processes, cellular components, and molecular functions. Protein pathways were annotated using the KEGG database (https://www.genome.jp/kegg/); Domain structural domain annotation was performed by InterProScan online service analysis (http://www.ebi.ac.uk/interpro/); WoLF PSORT (http://wolfpsort.org/) for subcellular localization analysis. *P* < 0.05 and the change of the multiplicity value of the difference was more than 2-fold as a significant up-regulation, and less than 1/2 as a significant down-regulation change criterion. Functional enrichment analysis was performed for the screened differentially acetylated modified proteins, and the *P*-value of the significant enrichment criterion conformed to the following criteria: GO enrichment analysis (*P* < 0.01); KEGG enrichment analysis (*P* < 0.01); and structural domain (Domain) enrichment analysis (*P* < 0.01). The motif-x software analyzed the sequences of specific acetylated amino acid peptides (10 amino acids upstream and downstream of the Kac site). NetSurfP software performed secondary structure analysis of differentially acetylated proteins, and the localization of acetylation sites revealed different secondary structures, including α-helices, β-turns, and random coils. STRING database (http://www.string-db.org/) was used for differential acetylated protein interaction analysis. Cytoscape software analyzed and visualized protein interactions in different biological processes. Differential acetylated proteins were clustered and analyzed based on the associations and differences present in specific functions (GO, KEGG, etc.).

### Plasmid constructions

The PMD19-T-BDH1 plasmid was precloned from the laboratory, and ligated to the pcDNA3.1–5xFlag vector using T4 ligation to construct the pcDNA3.1–5xFlag-BDH1 eukaryotic overexpression vector, which was used as a template for fixed-point mutation to construct pcDNA3.1–5xFlag-BDH1 (K91R), pcDNA3.1–5xFlag-BDH1(K91Q), pcDNA3.1–5xFlag-BDH1(K116R), pcDNA3.1–5xFlag-BDH1(K116Q), pcDNA3.1–5xFlag-BDH1(K122R), and pcDNA3.1–5xFlag-BDH1(K122Q) eukaryotic overexpression vectors. The mutations were confirmed by Sanger DNA sequencing, and the sequences of all primers used in this study are listed in Supplementary Table S1.

### Cell culture and treatment

The cells used in this experiment study were pre-preserved primary GMECs and HEK293T cells (Procell, Wuhan, China) from the laboratory, which were identified and tested for mycoplasma contamination. The GMECs were treated in the configuration of a cell line containing 10% FBS (Bio-Channel, Shanghai, China), 1% penicillin-streptomycin (Procell, Wuhan, China), 5 μg/mL hydrocortisone (Sigma-Aldrich, USA), 10 ng/mL EGF (Invitrogen, Carlsbad, CA, USA), 5 μg/mL insulin (Sigma-Aldrich, USA), and 90% DMEM-F12 (Gibco, New York, USA) were cultured in medium. HEK293T cells were cultured in medium supplemented with 10% FBS, 1% penicillin-streptomycin (Procell, Wuhan, China) and 90% DMEM (Gibco, New York, USA). All cell cultures were grown in a cell culture incubator at 37°C and 5% CO2. Cell transfection assays were performed according to the instructions of Lipofectamine2000 (Invitrogen, Carlsbad, CA, USA). The cells were transfected with pcDNA3.1–5xFlag-BDH1 (treatment group) plasmid and pcDNA3.1–5xFlag plasmid (control group), respectively. 16 h later, the solution was changed, and the deacetylase inhibitors were added to the wells transfected with pcDNA3.1-Flag-BDH1 plasmid with the final concentration of 50 nM of TSA (Selleck Chemicals, Houston, USA), NAM (TargetMol, Shanghai, China) at a final concentration of 100 uM, and the cells were collected after 12 h. The cells were transfected with the plasmids of the treated and control groups into the cells, and the solution was changed at 24 h. MG132 (CST, MA, USA) at 50 nM was added, and the cells were collected after 6 h.

### Immunoprecipitation(IP)

IP assay was performed on the cells collected after treatment and lysed by adding IP cell lysis buffer (Beyotime, Shanghai, China) containing PMSF (Beyotime, Shanghai, China) to each well, lysates were rotated and incubated for 30 mi at 4°C, and then centrifuged for 15 min at 4°C, 12,000xg. A total of 30 μL of the supernatant was taken and mixed with 10 μL 4x SDS buffer (Beyotime, Shanghai, China) for total protein analysis; the remaining supernatant was mixed with pre-washed ANTI-Flag M2 Affinity beads (Sigma-Aldrich, USA) and incubated at 4°C for 4 ~ 6 h. The immunoprecipitates were washed and then added to a 2x SDS buffer (Beyotime, Shanghai, China), and immunoblotting analysis was performed after denaturation at 95°C.

### Western blot(WB)

Protein samples were lysed by RIPA lysis buffer (Solarbio, Beijing, China) containing PMSF (Beyotime, Shanghai, China) on ice, quantified by BCA kit (#23225; Thermo Fisher Scientific, USA) and aliquoted, separated by SDS-PAGE and then transferred to PVDF membranes (Merck Millipore, Burlington, MA, USA). After 5% skimmed milk blocking, anti-acetylated lysine antibody (1:1,000, CST, MA, USA) and DDDDK Tag Recombinant Rabbit mAb (1:1,000, Diagbio, Hangzhou, China) were incubated overnight at 4°C, followed by room temperature incubation of HRP secondary antibody (1:4,000, CWBIO, Beijing, China) for 1 h, and developed using ECL Chemiluminescent Reagent (Thermo Fisher Scientific, USA) on a QuickChemi 5200 Chemiluminescent Imaging System (Monad Biotech, Wuhan, China).

### Mass spectrometry identification

The pcDNA3.1-Flag-BDH1 was transfected into HEK293T cells for 36 h and treated with deacetylase inhibitor (Med Chem Express, Shanghai, China) overnight. Subsequently, immunoprecipitates were separated by SDS-PAGE, followed by SYPRO Ruby staining, excision of approximately 40 kDa BDH1 protein bands, tryptic digestion with gel curd, and analyzed by HPLC-MS/MS.

### Triglyceride (TAG) content assay

The pcDNA3.1–5xFlag-BDH1, K116R, and K116Q plasmids (n = 3) were transfected into GMECs, respectively, and the triglyceride content was determined 48 h after transfection using the Triglyceride kit (Applygen, Beijing, China), with data collected by a BioTek zymography (Winooski, VT, USA). Triglyceride concentrations were normalized with the BCA Protein Assay Kit and expressed as μg/mg protein.

### BODIPY staining

The pcDNA3.1–5xFlag-BDH1, K116R and K116Q plasmids (n = 3) were transfected into GMECs, respectively. After 48 h, the cells were fixed in 4% paraformaldehyde at 4°C for 30 min, and 300 μL of BODIPY 493/503 staining solution (Invitrogen, Carlsbad, CA, USA; PBS 1: 1000 dilution) was added to each well for 30 min at room temperature, followed by 200 μL of DAPI staining solution (Beyotime, Shanghai, China) for 10 min. Lipid droplet imaging was captured by a cellular imaging reader (BioTek Instruments, Winooski, VT), and lipid droplet content was expressed as BODIPY fluorescent intensity (DAPI normalized) was expressed.

### Quantitative real-time PCR (RT-qPCR)

The pcDNA3.1–5xFlag-BDH1, K116R and K116Q plasmids (n = 3) were transfected into GMECs, and the total RNA was extracted by Trizol (Invitrogen, Carlsbad, CA, USA) after 24 h, the purity and concentration of the total RNA samples were quantified by Nanodrop 2000 Spectrophotometer (Thermo Fisher Scientific, MA, USA), and the samples were reverse transcribed into cDNA using the Prime-RiskScript RT kit (Takara, Shiga, Japan). The RT-qPCR assay was performed using ArtiCanCEO SYBR qPCR Mix (Tsingke Biotech, Beijing, China). UXT was used as an internal control to quantify and standardize the results, and 2^−AACT^ values were used for comparative quantification. Quantitative primer sequences are shown in Supplementary Table S2.

### Statistical analysis

Experimental data were expressed as mean ± standard error of measurement (mean ± SEM) of at least three independent experiments. Statistical analysis was performed using GraphPad Prism 9.0 software, and differences between groups were assessed by two-tailed unpaired Student t-test or one-way analysis of variance (ANOVA), with statistical significance defined as *P* < 0.05, **P < 0.01 and *P < 0.001.

## Results

### Quantitative acetylome and proteome analyses in mammary gland tissues of dairy goats

**Figure 1:**
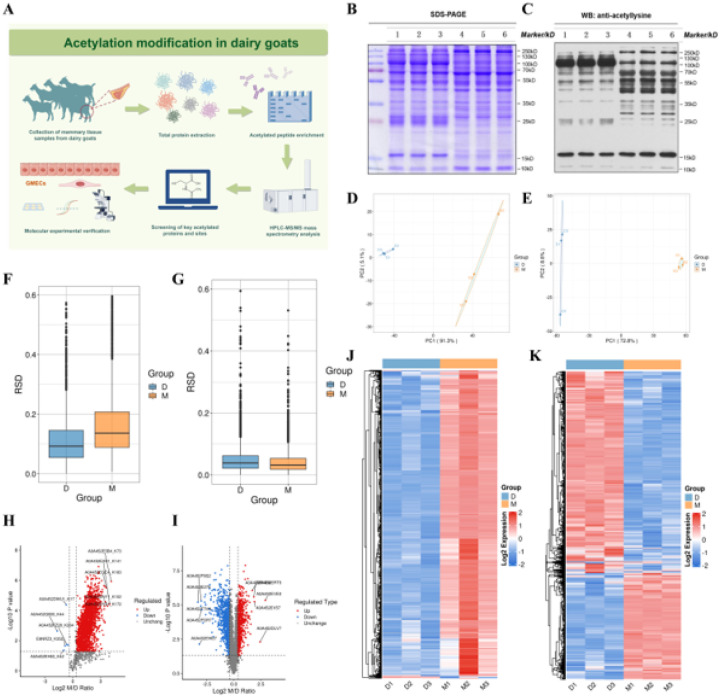
Acetylation modifications and proteomic analysis in different lactation cycles. (A) Experimental design flow chart. (B) Gel electrophoresis with Coomassie Brilliant Blue staining (total protein volume: 20ug). Lanes 1–3: total protein samples from mammary tissue of dairy goats during dry milk period (D1-D3); Lanes 4–6: total protein samples from mammary tissue of dairy goats during peak lactation period (M1-M3). (C) WB results of anti-pan acetylated lysine antibody. The lane labelling was the same as A. (D) PCA plots of the quantification of modification sites between samples from each group of the acetylation modification group (D: dry milk stage, M: peak lactation). (E) PCA plots of the quantification of proteins between samples from each group of the proteins group (grouping was the same as D). (F) Box line plots of the RSD of the quantification values of modification sites between samples from each group of the acetylation modification group (grouping was the same as D). (G) Box line plot of the quantification values of proteins between Box line plots of RSD of quantitative values of proteins between samples (grouped as in D). (H) Volcano plots of differential modification sites between samples in each group of the acetylation modification group (Screening criteria: Foldchange (M/D) > 1.5; P-values were determined by two-tailed t-tests, *P* < 0.05). Red dots indicate that differential modification sites are significantly different up-regulated in the treatment group relative to the control group, blue dots indicate significantly different down-regulated, and grey indicates no significant difference. (I) Volcano plot of differential proteins among samples from each group of the proteome (same criteria as H). Red dots indicate significant difference up-regulation of differential proteins in the treatment group relative to the control group, blue dots indicate significant difference down-regulation, and grey indicates no significant difference. (J) Heatmap analysis of the expression pattern of differential modification sites between samples in each group of the acetylation modification group (pink represents strong enrichment, blue represents weak enrichment). (K) Heatmap analysis of the expression pattern of differential proteins between samples in each group of the proteins group (coloring scheme as in J).

To explore the role of acetylation modification in regulating mammary gland function in dairy goats, we designed the following experiment (Fig. 1A). we first performed WB to detect total protein levels in mammary gland tissues collected from two lactation periods (peak lactation vs. the dry period). All samples displayed clear protein bands with no signs of degradation, suggesting high sample quality (Fig. 1B). Subsequent detection using a pan-anti-acetylated lysine antibody (Fig. 1C) showed significant differences in electrophoretic migration between the two groups, indicating that acetylation modification may play an pivotal role in the mammary gland across different lactation periods. To evaluate data reliability, principal component analysis (PCA) and standard deviation coefficient (RSD) analyses were conducted. As shown in Fig. 1D–G, samples from peak lactation and dry periods showed distinct clustering in both quantitative acetylomic and proteomic datasets, indicating high reproducibility and reliability across biological replicate. Differential expression analysis of the acetylome revealed that 856 proteins were up-regulated and 6 proteins were down-regulated in the peak lactation group compared to the dry period (Fig. 1H). In the corresponding proteomic analysis, 527 proteins were up-regulated and 991 proteins were down-regulated (Fig. 1I). Heat map analysis further confirmed that the acetylation modification sites were generally enriched in lactation samples (Fig. 1J and K).

### Characterization of lysine acetylation modifications in mammary gland tissues of dairy goats

**Figure 2:**
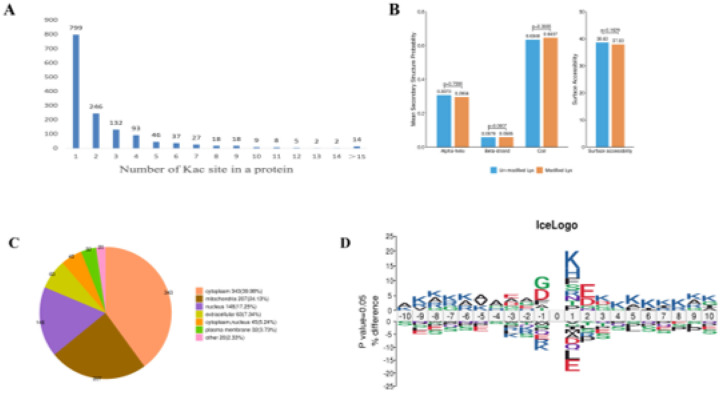
Acetylation modification site distribution and preference analysis. (A) Distribution of the number of lysine acetylation sites in each modified protein. (B) Distribution of lysine in the secondary structure region of proteins. (C) Prediction of subcellular localization of lysine proteins. (D) Preferred motif analysis map of acetylation modification sites.

We further analyzed lysine acetylation (Kac) modification profiles in mammary gland tissues of dairy goats. Quantitative acetylome analysis showed that individual proteins harbored between 1 and 26 Kac sites. Among these, 799 proteins contained only one Kac site, whereas 657 proteins contained two or more Kac sites (Fig. 2A). These findings confirm the successful construction of a comprehensive acetylation-modified proteome for dairy goat mammary tissue. Structural analysis of acetylated proteins using NetSurfP showed that approximately 30% of Kac sites were located within α-helical structural domain, 6% within the β-folded structural domain, and the remaining 64% in irregularly coiled regions (Fig. 2B). Subcellular localization analysis revealed that acetylated proteins were primarily distributed in the cytoplasm (39.98%), mitochondria (24.13%), nucleus (17.25%) and extracellular space (7.34%) (Fig. 2C). Finally, we counted the sequence preferences on both sides (± 10 amino acids) of all identified Kac sites. This analysis revealed significant sequence enrichment features around the acetylation sites (Fig. 2D), with lysine (K) residues occurring most frequently at the - 9 to −6, + 1, and +3 to + 10 sites.

### Functional enrichment analysis of differentially acetylated proteins and differential proteins in dairy goats

**Figure 3:**
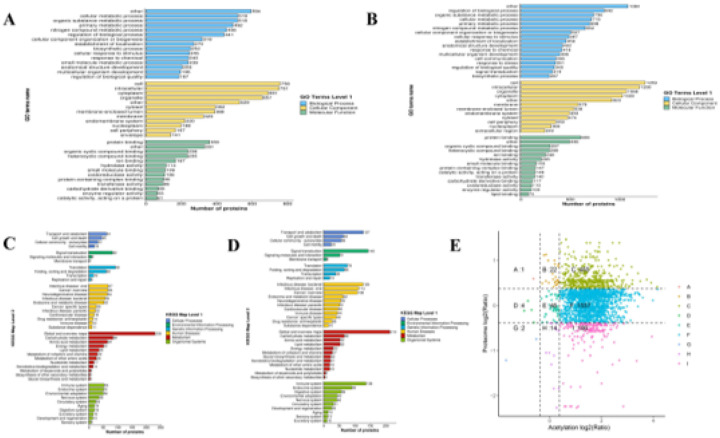
Functional enrichment characteristics of differentially acetylated proteins and expressed proteins. (A) GO enrichment analysis of differentially acetylated proteins in dairy goats. (B) GO enrichment analysis of differentially expressed proteins in dairy goats. (C) KEGG enrichment analysis of differentially acetylated proteins in dairy goats. (D) KEGG enrichment analysis of differentially expressed proteins in dairy goats. (E) Nine-quadrant diagram of differentially acetylated proteins and differentially expressed proteins in dairy goats.

To investigate the functional properties of differentially acetylated proteins (DAPs) and differentially expressed proteins (DEPs), we performed a systematic functional enrichment analysis. GO analysis showed that DAPs were significantly enriched in cellular metabolism, organic matter metabolism, primary metabolic processes, and metabolism of nitrogen compounds in biological processes. In terms of cellular component classification, DAPs were primary localized to cellular, intracellular, cytoplasmic and cytosolic organelles. Regarding molecular functions, they were predominantly associated with protein-binding, organic cyclic compound-binding, and heterocyclic compound-binding (Fig. 3A). Similarly, GO enrichment analysis of DEPs showed significantly enriched in bioregulation, organic matter metabolism, cellular metabolism and primary metabolism. The cellular component distributions of DEPs was also consistent with that of DAPs, including cellular, intracellular, organelle, and cytoplasmic compartments. Molecular functions were likewise dominated by protein-binding and interactions with cyclic compounds (Fig. 3B). KEGG pathway analysis further revealed that DAPs were significantly involved in pathways related to signal transduction, membrane transport, translation, amino acid metabolism, energy metabolism and lipid metabolism (Fig. 3C). In contrast, DEPs were mainly enriched in catabolic, signal transduction, translation and immune-related pathways (Fig. 3D). Through the nine-quadrant correlation analysis integrating acetylome and proteomic data (Fig. 3E), we identified 455 proteins that were both differentially acetylated and differentially expressed, suggesting a potential synergistic regulatory association between lysine acetylation and protein expression in the mammary gland of dairy goats.

### BDH1 acetylation regulates milk fat biosynthesis in dairy goats

**Figure 4:**
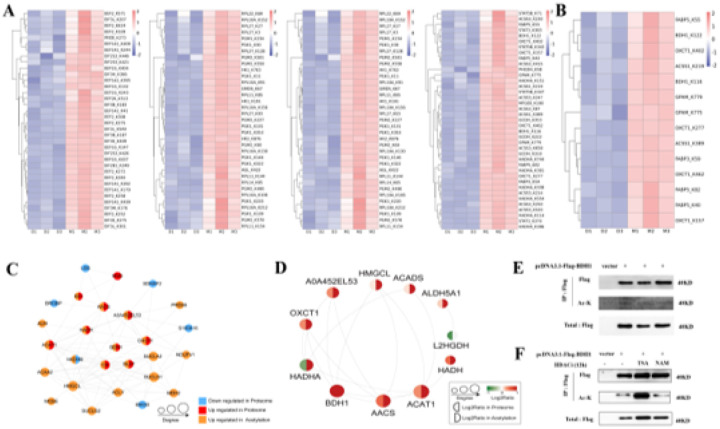
Validation analysis of acetylation modification of BDH1 protein in dairy goats. (A) Heat map related to each pathway of differentially acetylated modified proteins in dairy goats: lactation synthesis, lactose synthesis, milk protein synthesis, and milk fat synthesis. (B) Cluster analysis map of differentially acetylated modified proteins. (C) Interaction network map of differentially acetylated modified proteins of BDH1. (D) Interaction network map of the pathways of differentially acetylated modified proteins of BDH1. (E) Transfection of the pcDNA3.1-5×Flag -BDH1 plasmid after transfection, BDH1 acetylation modification was detected by IP and WB (using anti-acetylated lysine pan antibody). (F) Cells transfected with pcDNA3.1-Flag-BDH1 plasmid were treated with 50 nM TSA or 100 μM NAM for 12 h, respectively, and BDH1 acetylation levels were detected by IP and WB.

By integrating data on milk component synthesis pathways with acetylation modification profiles from mammary gland tissues, we identified 54 DAPs related to milk component synthesis. These included 17 key proteins that regulate milk protein synthesis, such as MTOR and BCAT2; 13 key proteins involved in milk fat synthesis, such as ACSS1 and BDH1; 12 key proteins that affect lactation, such as EEF1A1 and EEF2; as well as 12 key proteins involved in lactose synthesis, such as RPL10A and PGK1. Heatmap analysis (Fig. 4A) revealed that these proteins exhibited significant elevated levels of acetylation during the lactation period compared to the dry period (site-specific data are provided in Appendix 1). Further focusing on the milk fat synthesis pathway, 6 core proteins, including FABP5 and BDH1, were identified through screening of the milk fat regulatory network. Cluster analysis confirmed that these proteins exhibit highly synergistic acetylation modifications (Fig. 4B). In-depth validation of the key protein BDH1 revealed that its protein-protein interaction (PPI) network (Fig. 4C) directly interacts with lipid metabolism regulatory factors such as AACS and OXCT1, while pathway enrichment (Fig. 4D) is significantly associated with ketone body synthesis and metabolism. In addition, cell experiments demonstrated that HEK293T cells transfected with Flag-BDH1 exhibited acetylation bands detected by IP-WB (Fig. 4E), and TSA (HDAC inhibitor) treatment significantly enhanced its acetylation levels (Fig. 4F), revealing that the HDACs family is the core regulatory factor for BDH1 deacetylation. In summary, BDH1 regulates ketone metabolism pathways through acetylation modification, thereby influencing milk fat synthesis in dairy goats. Statistical significance values for each DAP and site are detailed in Supplementary Table 3.

### Acetylation level at K116 modulates BDH1 protein stability in HEK293T cells

**Figure 5:**
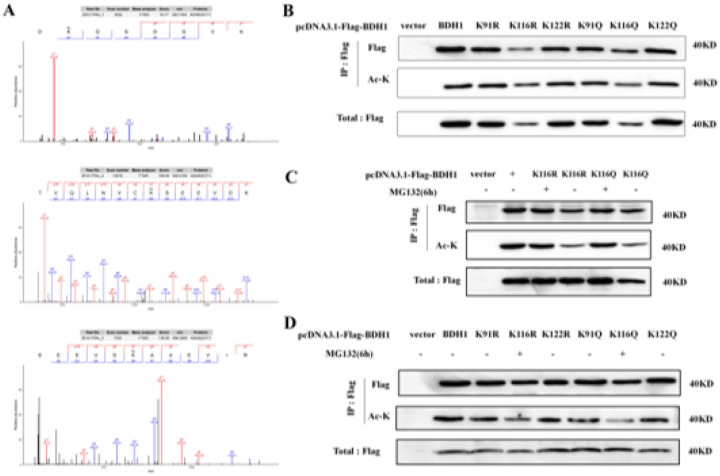
Identification of the acetylation site of BDH1 protein in dairy goats and its effect on protein stability. (A) Flag-BDH1 was immunoprecipitated from HEK293T cells treated with HDAC inhibitor, and 40 kDa bands were separated and cut by SDS-PAGE for in-gel trypsin digestion, and the presence of acetylation modification at the K91, K116 and K122 sites was confirmed by LC-MS/MS analysis. (B) After transfecting HEK293T cells with the constructed mutant vectors of K91, K116 and K122 Q/R and wild type respectively, WB assay revealed that the mutation of K116 caused protein degradation of the structure of BDH1 protein. (C) The K116Q/R mutant was transfected into HEK293T cells and treated with the proteasome inhibitor MG132 (50nM, 6h), and the WB assay showed that the protein degradation was effectively reversed, demonstrating that the degradation pathway was proteasome-dependent. (D) After transfecting HEK293T cells with the constructed mutant vectors of Q/R at K91, K116 and K122 sites and the wild type respectively, and comparing the protein levels of all the mutants before and after treatment with MG132, the WB analysis confirmed that only the acetylation status of the K116 site (Q/R) directly regulated the stability of BDH1 through the proteasome pathway, and the K91/K122 mutation had no significant effect.

Using LC-MS/MS analysis (Fig. 5A), we identified 3 lysine acetylation sites, K91, K116, and K122, on the BDH1 protein in dairy goats. To investigate their functions, we constructed BDH1 mutants simulating a highly acetylated state (K→Q mutation) and a deacetylated state (K→R mutation). Cell experiments revealed that the K116 site mutation (K116Q/R) led to significant degradation of the BDH1 protein (Fig. 5B), and treatment with the proteasome inhibitor MG132 effectively blocked the degradation of the K116Q/R mutant (Fig. 5C). Further validation via IP-WB (Fig. 5D) confirmed that K116 is the core acetylation modification site of BDH1, and the acetylation level at this site directly regulates BDH1 protein stability.

### Acetylation of BDH1 at K116 directly modulates lipid biosynthesis in GMECs

**Figure 6:**
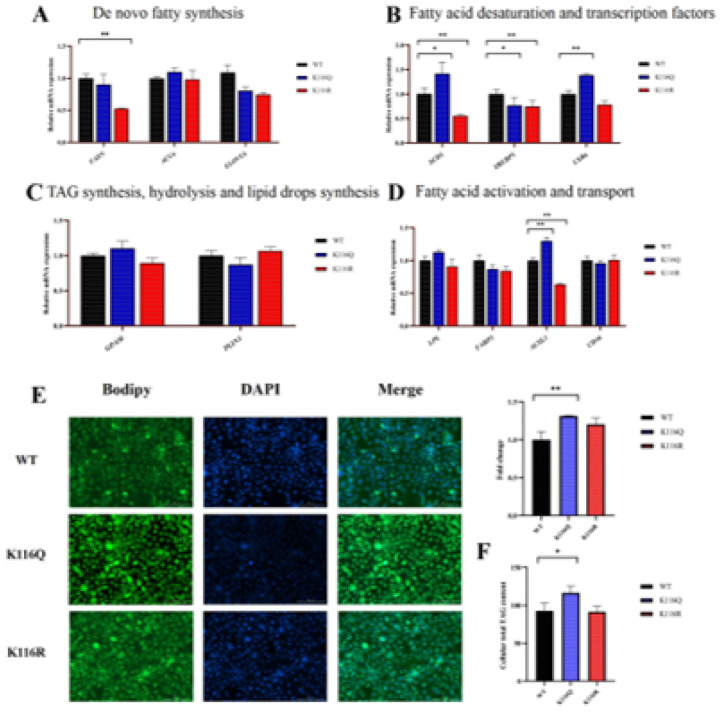
Acetylation modification at the K116 site of BDH1 protein promotes lipid droplet accumulation and triglyceride synthesis in GMECs. (A-D) After transfection of pcDNA3.1–5×Flag-BDH1 (wild-type), K116R, or K116Q plasmids for 24 h, RT-qPCR was performed to detect the effects of acetylation/deacetylation at the K116 site of BDH1 on the expression of the expression of the following genes: (A) genes related to fatty acid synthesis from scratch; (B) genes related to fatty acid desaturation and transcriptional regulation; (C) genes related to triglyceride synthesis, hydrolysis and lipid droplet formation; and (D) genes related to fatty acid activation and transport. (E) Lipid droplet accumulation in GMECs was observed by BODIPY staining 48 h after transfection with pcDNA3.1–5×Flag-BDH1 (wild type), K116R or K116Q plasmids. (F) After transfection of pcDNA3.1–5×Flag-BDH1 (wild type), K116R or K116Q plasmids for 48 h, the intracellular TAG-containing data were detected using a commercial kit and expressed as mean ± standard error of the mean (n = 3 biological replicates), and the differences between the groups were statistically analyzed by two-tailed Student t-test or one-way analysis of variance (ANOVA),* *P*< 0.05, **P < 0.01 and *P < 0.001 indicate statistical significance.

To elucidate the role of acetylation modification at the BDH1 K116 site in the regulation of lipid metabolism in GMECs, functional studies were performed by transfection of an acetylation mimic mutant (K116Q) and a deacetylation mimic mutant (K116R). RT-qPCR analyses showed that (Fig. 6A–D), compared with the K116R group, the K116Q group significantly up-regulated the expression levels of lipid metabolism-related genes *SCD1, LXR* and *ACSL1* expression levels and restored the expression of *SREBP1* while the K116R group significantly suppressed the expression of *SCD1, FASN, SREBP1* and *ACSL1*. Further phenotypic analyses showed (Fig. 6E–F) that K116Q transfection significantly increased intracellular lipid droplet content and TAG level, and these results fully demonstrated that acetylation modification of the BDH1 K116 site plays a key role.

## Discussion

Lysine acetylation, a key post-translational modification, has long been the focus of functional studies on histones. However, recent advances have revealed that lysine acetylation occurs widely in non-histone proteins, such as transcription factors, signaling molecules, metabolic enzymes, and plays a central regulatory role in various cellular processes, including apoptosis, signaling, membrane stability, and others [16–19]. Innovations in mass spectrometry and acetylated peptide enrichment methods have greatly facilitated the study of non-histone acetylation, particularly in the field of metabolic regulation [20, 21].

This study integrates TMT labeling, acetylated peptide enrichment, and HPLC-MS/MS techniques to quantify the acetylation modification proteome for the first time in mammary tissues of Saanen dairy goats during peak lactation and dry milking stages. We identified several differentially acetylated proteins and sites that were significantly regulated by lactation stage, with more up-regulated acetylation events observed during lactation. This finding suggests that lysine acetylation plays a role in regulating the biological process associated with lactation in dairy goats.

Secondary structure analysis of the acetylation sites revealed that approximately 30% of acetylation sites were located in α-helices, 6% in β-sheets, and 64% in randomly convoluted regions. Motif analysis indicated that lysine residues with specific surrounding amino acid sequences are more susceptible to acetylation, which may reflect the sequence preferences of acetyltransferases for substrates, analogous to the mechanism of kinase phosphorylation site recognition [22]. Subcellular localization revealed that differentially acetylated proteins were predominantly distributed in the cytoplasm (39.98%) and mitochondria (24.13%), with fewer in the nucleus (17.25%), This distribution pattern closely aligns with acetylome profiles reported in human and mouse liver tissues [23]. Acetylation modifications can alter the charge and conformation of proteins, thereby affecting their subcellular localization. For instance, acetylation of glyceraldehyde-3-phosphate dehydrogenase (GAPDH) has been shown to induce its translocation from the cytoplasm to the nucleus [24]. Further functional enrichment analyses demonstrated that although DAPs and DEPs highly overlapped in subcellular localization (e.g., cytoplasm and organelles) and molecular functions (e.g., protein binding), they showed distinct functional roles. DAPs were significantly enriched in signal transduction, membrane transport and amino acid/lipid metabolism pathways, suggesting that acetylation modification serves as a rapid response mechanism to precisely regulate metabolic fluxes in the lactating mammary gland either by directly modifying metabolic enzyme activities or altering their subcellular localization. In contrast, DEPs were mainly enriched in processes related to bioregulation, catabolism and immune pathways.

Critically, integrative analysis identified 455 proteins that were both differentially acetylated and differentially expressed, suggesting extensive crosstalk between acetylation and protein abundance. This interplay may regulate protein stability or modify transcription factor activity to affect gene expression, thereby coordinating mammary gland function at both the transcriptional and translational levels. Cluster analysis identified 54 key acetylated proteins that were potentially involved in regulating milk protein, milk fat, lactose synthesis and overall lactation. These include MTOR, BCAT2, GOT1/2, BDH1, ACSS1, STAT5B, FABP5, and GPAM. mTOR is a core regulator of milk protein synthesis and functions by phosphorylating the downstream targets S6K1 and 4E-BP1 [25]. ACSS1, STAT5B, FABP5 and GPAM are critical for milk lipid synthesis, participating in de novo fatty acid synthesis, uptake, transport, desaturation, triglyceride formation, and lipid droplet assembly [26].

Previous studies have found that nearly all metabolic enzymes involved in major metabolic pathways, such as glycolysis, gluconeogenesis, the tricarboxylic acid cycle, the urea cycle, fatty acid metabolism, and glycogen metabolism, undergo acetylation modifications [27, 28]. Building on this, our study focused specifically on the potential function of acetylation modification of BDH1 in lipid metabolism in mammary epithelial cells of dairy goats. BDH1, a member of the short-chain dehydrogenase/reductase (SDR) family and a key enzyme in ketone body metabolism [29, 30], reversibly catalyzes the β-hydroxybutyrate (BHBA) interconversion with acetoacetate (AcAc) [31]. Its important roles in cardiac energy metabolism [32], hepatic ketone body production, and lipid homeostasis have been well documented [33, 34, 35]. Increasing evidence also suggests that BDH1 is involved in milk lipid synthesis processes in the mammary gland [36–39]. Importantly, BDH1-mediated BHBA/AcAc homeostasis is essential for acetyl coenzyme A-dependent acylation modifications, including histone acetylation and β-hydroxybutyrylation [40, 41]. Notably, BHBA itself functions as an endogenous HDAC inhibitor [42], and BDH1 overexpression has been shown to increase histone acetylation levels in the cardiac tissue [43]. While the metabolic role of BDH1 and its metabolites in epigenetic regulation is increasingly recognized, the impact of BDH1’s own post-translational modifications, especially acetylation, on its function in mammary lipid metabolism remains largely unexplored. In this study, we investigated the role of BDH1 acetylation in regulating lipid metabolism in dairy goat mammary epithelial cells using the broad-spectrum HDAC inhibitor TSA with the Sirtuin inhibitor NAM [44, 45]. TSA significantly elevated BDH1 acetylation levels, suggesting that members of the HDACs family are involved in BDH1 deacetylation Regulation.

Lysine acetylation can alter a protein’s charge distribution and spatial conformation, enhancing its interaction with other molecules. This modification often intersects with ubiquitination to co-regulate biological processes [46, 47]. For example, Butler demonstrated that acetylation blocks ENaC channel ubiquitination, stabilizing their membrane localization [48], and Gudiksen et al found that phosphorylation and acetylation synergistically regulate PDC complex activity [49]. In our study, both K116Q and K116R mutants of BDH1 underwent protein degradation, which was reversed by the proteasome inhibitor MG132. This suggests that BDH1 acetylation may activate the ubiquitin-proteasome degradation pathway, similar to the regulatory mechanism of p53 [50]. We conducted functional studies in goat mammary epithelial cells by simulating the acetylation (K116Q) and deacetylation states (K116R) of BDH1. The acetylation status significantly affected BDH1’s ability to regulate the expression of lipid synthesis-related genes. Specifically, the K116Q mutant appeared to suppress lipid synthesis by down-regulating the expression of *LXRα* and *ACSL1* genes. In contrast, the K116R mutant more strongly inhibited lipid synthesis by downregulating the expression of *SCD1, FASN* and *ACSL1* genes. Interestingly, both mutants significantly decreased expression of the core lipid synthesis transcription factor *SREBP1*, though the K116Q mutant partially restored *SREBP1* expression compared to K116R, suggesting a nuanced regulatory role of BDH1 acetylation at this critical node. These transcriptional changes were preliminarily supported by phenotypic data, including reduced triglyceride content and lipid droplet accumulation, collectively indicating that the acetylation/deacetylation status of BDH1 plays a significant role in regulating lipid metabolism processes in mammary epithelial cells of dairy goats.

## Conclusions

The first comprehensive acetylome profiles of mammary gland tissues from dairy goats were successfully established using HPLC-MS/MS, and BDH1 was identified as a key acetylated protein that varies across different lactation stages. BDH1 was found to be acetylated at lysine 116 (K116) site, with members of the HDACs family identified as primary regulators of its deacetylation. Functional studies demonstrated that the acetylation status of BDH1 significantly affects lipid biosynthesis in GMECs. These findings provide new insights into the molecular mechanisms by which post-translational modifications contribute to the regulation of lactation and the improvement of goat milk quality.

## Supplementary Material

Supplementary Files

This is a list of supplementary files associated with this preprint. Click to download.

- Supplementarymaterial.docx
- GraphicalAbstract.docx

## Data Availability

All data are available from the corresponding author upon request.
